# Effect of Vitamin D Supplementation on Depression in Adults: A Systematic Review of Randomized Controlled Trials (RCTs)

**DOI:** 10.3390/nu15040951

**Published:** 2023-02-14

**Authors:** Dominika Guzek, Aleksandra Kołota, Katarzyna Lachowicz, Dominika Skolmowska, Małgorzata Stachoń, Dominika Głąbska

**Affiliations:** 1 Department of Food Market and Consumer Research, Institute of Human Nutrition Sciences, Warsaw University of Life Sciences (WULS-SGGW), 159C Nowoursynowska Street, 02-776 Warsaw, Poland; 2 Department of Dietetics, Institute of Human Nutrition Sciences, Warsaw University of Life Sciences (WULS-SGGW), 159C Nowoursynowska Street, 02-776 Warsaw, Poland

**Keywords:** vitamin D, supplementation, supplement, depression, depressive symptoms, major depressive disorder, diet therapy, Randomized Controlled Trials (RCTs)

## Abstract

Vitamin D is a nutrient potentially beneficial in the treatment of depression. The study aimed to carry out a systematic review of the studies assessing the influence of vitamin D supplementation on depression within Randomized Controlled Trials (RCTs). The systematic review was prepared on the basis of the Preferred Reporting Items for Systematic Reviews and Meta-Analyses (PRISMA) guidelines, and was registered in the International Prospective Register of Systematic Reviews (PROSPERO) database (CRD42020155779). The peer-reviewed studies available within PubMed or Web of Science databases until September 2021 were taken into account. The number of screened records was 8514, and 8 records were included. Two independent researchers conducted screening, including, reporting, and risk of bias assessment using the revised Cochrane risk-of-bias tool for randomized trials. The included studies presented a population of patients with major depressive disorders or general depression, as well as bipolar depression or postpartum depression. The majority of included studies were conducted for 8 weeks or 12 weeks, while one study was conducted for 6 months. Within the large number of included studies, a daily dose of 1500 IU, 1600 IU, or 2800 IU was applied, while within some studies, a vitamin D dose of 50,000 IU was applied weekly or biweekly. Among applied psychological measures of depression, there were various tools. In spite of the fact that the majority of included studies (five studies) supported the positive effect of vitamin D supplementation for the psychological measure of depression, for three studies the positive influence was not supported. A medium risk of bias was indicated for six studies, while a high risk of bias was defined for only two studies, due to deviations from the intended interventions and in measurement of the outcome, as well as for one study, also arising from the randomization process and due to missing outcome data. Based on conducted assessment, it should be emphasized that there are only four studies supporting the positive influence of vitamin D supplementation for the psychological measure of depression of the medium risk of bias, while two studies of a medium risk of bias did not support it. Taking this into account, the conducted systematic review is not a strong confirmation of the effectiveness of vitamin D supplementation in the treatment of depression.

## 1. Introduction

Depression is defined as a mood disorder causing a persistent feeling of sadness and loss of interest [1], while the depressive disorders are classified as follows: disruptive mood dysregulation disorder, major depressive disorder, persistent depressive disorder (dysthymia), premenstrual dysphoric disorder, and depressive disorder due to another medical condition [2]. The diagnosis of depression is based on assessment of nine depressive symptoms (sleeping disturbance, reduction in interest/pleasure, guilt feelings/thoughts of worthlessness, energy changes/fatigue, impairment of concentration/attention, appetite/weight changes, psychomotor disturbances, suicidal thoughts, and depressed mood), while in order to diagnose depression, five of them must be present—including depressed mood or interest/pleasure reduction [1]. The etiology of depression includes stressful experiences which may trigger depression in vulnerable patients, while individual susceptibility results from biological and psychosocial characteristics and circumstances combined [3].

The study of the prevalence of depression conducted in communities from 30 countries for 20 years indicated the one-year and lifetime prevalence of depression of 7.2% and 10.8%, respectively, being higher for women than men [4]. At the same time, the meta-analysis of community-based studies assessing the prevalence of depression during the COVID-19 outbreak revealed a pooled prevalence of 25%, being seven times higher than a global prevalence of depression in 2017, estimated as 3.44% [5]. It is a really serious public health problem, as the World Health Organization (WHO) indicated a higher risk of premature death for individuals with depression, amounting to 40–60% for individuals with a major depressive disorder, which results from physical health problems that are often left unattended and from suicides [6].

The recommended treatment of depression includes psychological treatments, medication, and general measures such as relaxation techniques; they are often combined in order to obtain a better effect [7]. Moreover, the meta-analysis of Randomized Controlled Trials (RCTs) by Firth et al. [8] indicated that dietary interventions may be effective in reducing symptoms of depression across the population and in spite of the fact that little is known about necessary components of effective dietary intervention to improve mental health—nor about the mechanism—diet can already be defined as a promising therapeutic intervention to accompany a standard therapy.

The studies of association between diet and mental health problems are based either on the effect of food products or on the effect of specific nutrients, while for depression there are indicated food products such as olive oil, fish, fruits, vegetables, nuts, legumes, poultry, dairy, and unprocessed meats, as well as nutrients such as calcium, chromium, zinc, magnesium, vitamin D, folate, vitamin B12, and polyunsaturated fatty acids [9]. Among the indicated nutrients, vitamin D is widely studied for its association with general mental health, while a majority of studies indicated that the main psychophysiological variables associated are depression and anxiety, followed by mood [10].

In spite of the fact that there are plenty of studies analyzing the association between vitamin D supplementation and course of depression, many systematic reviews and meta-analyses are quite old, and a number of them present contradictory results. Among systematic reviews and meta-analyses published until 2015, those by Gowda et al. [11] and by Li et al. [12] indicated that supplementation of vitamin D do not influence depression reduction, but those by Shaffer et al. [13] and by Spedding [14]—in studies defined as free from biological flaws—indicated that supplementation of vitamin D may effectively reduce symptoms in patients with diagnosed depression. Among systematic reviews and meta-analyses published recently, the study by Vellekkatt and Menon [15] assessed only major depressive disorder, while those by Mikola et al. [16] and Albuloshi et al. [17] assessed general depressive symptoms, but all of them indicated positive effect of vitamin D supplementation. However, it should be emphasized that the listed studies included either only major depressive disorder [15], being one of the known types of depression [18], or depressive symptoms [16,17], being a broader spectrum than the depression itself [1], which was associated with the number of RCTs included within the mentioned studies. At the same time, there are some recently retracted studies [19], which were previously included into mentioned systematic reviews and meta-analyses, so the retraction may change the results and conclusions.

Taking into account the promising results of the recent studies assessing the influence of vitamin D supplementation on major depressive disorder or depressive symptoms, the study aimed to carry out a systematic review of the studies assessing the influence of vitamin D supplementation on depression within RCTs.

## 2. Materials and Methods

### 2.1. Study Registration and Study Design

The literature searching, screening, inclusion, and reporting was based on the guidelines of the Preferred Reporting Items for Systematic Reviews and Meta-Analyses (PRISMA) [20]. The registration in the International Prospective Register of Systematic Reviews (PROSPERO) database was conducted (registration number CRD42020155779).

The peer-reviewed studies presenting results of RCTs, available within PubMed or Web of Science databases until September 2021 and published in English, were intended to be included. A systematic literature searching was conducted within two stages—for studies published until October 2019 (before COVID-19) and from October 2019 to September 2021 (after announcing the first COVID-19 case). For the studies included within the second stage, the additional searching of the COVID-19 incidence information in the studied group was conducted. The procedure applied was based on a previously adapted one for the assessment of vitamin D on mental health in children [21] and adults [22], including populations with diabetes [23], multiple sclerosis [24], as well as inflammatory bowel diseases and irritable bowel syndrome [25].

### 2.2. Eligibility Assessment and Inclusion/Exclusion Procedure

The studies assessing the influence of supplementation of vitamin D on depression within RCTs were intended to be included, based on the following inclusion criteria:

1. study conducted in adults;
2. studied population of patients with depression diagnosed;
3. study presenting oral vitamin D supplementation of known dose;
4. depression monitored within the study using a valid mental health outcome measure;
5. study described as RCT;
6. study published as an article in a peer-reviewed journal.

The exclusions were conducted based on the following exclusion criteria:

1. animal model study;
2. study presenting influence of multiple nutrients combined;
3. study conducted in subjects with any concurrent physical disease or disability;
4. study conducted in pregnant women;
5. study conducted in subjects with concurrent eating disorders;
6. study conducted in subjects with concurrent intellectual disabilities;
7. study not published in English.

The patient, intervention/exposure, comparator, outcome, and study design (PICOS) criteria are presented in Table 1.

### 2.3. Searching Procedure

The detailed electronic search strategy applied for the systematic review within PubMed or Web of Science databases is presented in Supplementary Table S1.

After searching databases for potentially eligible studies, duplicated records were removed, if found both within PubMed and Web of Science databases. Afterwards, potentially eligible studies were identified, while using inclusion and exclusion criteria. In order to identify eligible studies, the procedure was conducted within three stages: based on titles, based on abstracts, and based on full texts. Identification based on titles and based on abstracts were conducted using data available within PubMed and Web of Science databases. Only for the studies defined as potentially eligible, after a procedure based on title and based on abstract, the full texts were assessed. In order to obtain the full text of the study, electronic databases and libraries were searched, and if not available, the corresponding authors were asked for them. The identification within all stages was conducted by two researchers independently, but in case of disagreement, the third researcher was asked for an opinion.

**Figure 1:**
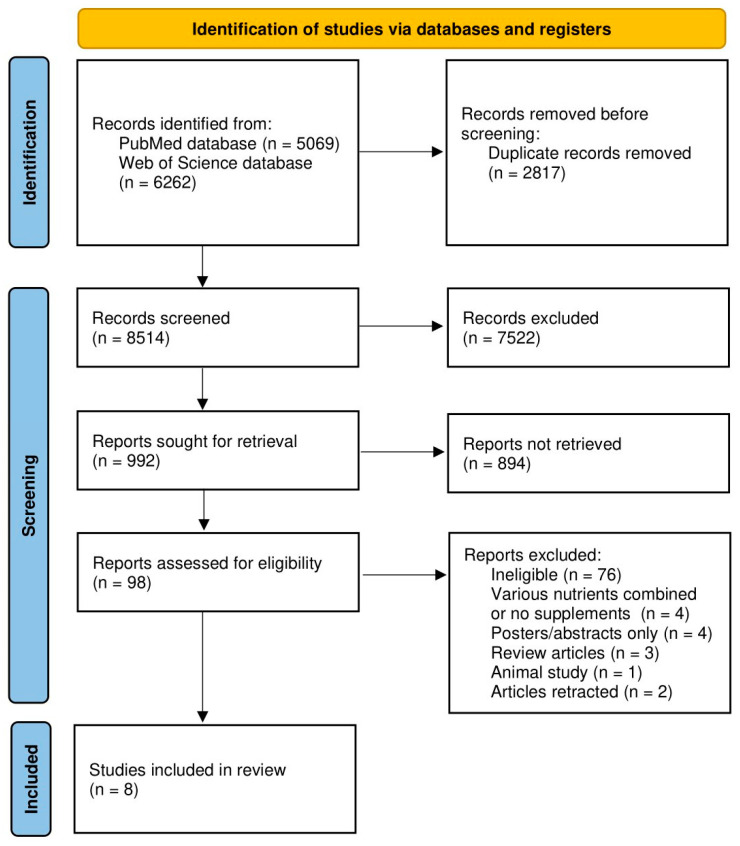
The procedure of identification, screening, eligibility assessment, and including studies.

The procedure of identification, screening, eligibility assessment, and including studies is presented in Figure 1.

### 2.4. Data Extraction Procedure and Study Assessment Procedure

After including eligible studies, they were analyzed in order to extract necessary data to describe the study and the influence of vitamin D supplementation on depression. The following data were extracted:

1. the general description of the study and studied population (including authors and year of the study; country/detailed location; studied population; period of the study);
2. the description of the studied population (including number of participants; female/male proportions; age; inclusion and exclusion criteria);
3. the description of the supplementation of vitamin D (including dosage regimen; intervention duration) and of the assessment of depression status (including psychological measure);
4. the observations and conclusions (based on those formulated by authors of the study).

If possible, all the data were obtained from a published study (or other publications referred within the study). If any information was missing, the corresponding authors were contacted. The data extraction was conducted by two researchers independently, but if any disagreement appeared, the third researcher was asked for an opinion.

The quality of the studies included was assessed based on the risk of bias defined for the studies [26]. The revised Cochrane risk-of-bias tool for randomized trials was used with the RoB 2 tool [27]. Each study was assessed within the following five domains of the risk of bias: arising from the randomization process, due to deviations from the intended interventions, due to missing outcome data, in measurement of the outcome, in selection of the reported result; and afterwards, it was assessed for the overall risk of bias [28].

## 3. Results

The general descriptions of the studies and studied populations within studies included to a systematic review [29,30,31,32,33,34,35,36] are presented in Table 2. The large number of included studies were conducted in Iran [29,32,33,34], while the others were conducted in Saudi Arabia [35], China [36], the United States of America [30], or Denmark [31]. They presented a studied populations of patients with major depressive disorders [29,35] or general depression [31,32,33,36], but also bipolar depression [30] or postpartum depression [34].

The descriptions of the studied populations within studies included to a systematic review are presented in Table 3. The included studies were conducted mainly in small to medium size samples of less than 100 participants (divided into studied groups and control groups) [29,30,31,32,33,34,35], while only one study was conducted in a larger group [36]. The included studies were conducted mainly in populations of young [34] to middle-aged adults [29,30,31,33,35], but one study was conducted in a group of older patients [32]. The inclusion and exclusion criteria were based on the studied population, with vitamin D deficiency sometimes indicated within inclusion criteria [30,36] or exclusion criteria [31], depending on the study.

The observations and conclusions formulated within studies included to systematic review are presented in Supplementary Table S2, and the descriptions of the vitamin D supplementation and the assessment of depression status accompanied by summary of conclusions formulated within studies included to a systematic review are presented in Table 4. The majority of included studies were conducted for 8 weeks [29,32,33,34] or 12 weeks [30,31,35], while one study was conducted for 6 months [36]. Within the large number of included studies, the daily dose of 1500 IU [29,30,31], 1600 IU [36], or 2800 IU was applied [31]. At the same time, within some studies, the vitamin D dose of 50,000 IU was applied weekly [32,35], or biweekly [33,34]. Among applied psychological measures of depression were various tools, such as the Beck Depression Inventory (BDI) [29,33,35], Hamilton Depression Rating Scale (HDRS) [29,36], Montgomery-Åsberg Depression Rating Scale (MADRS) [30], Hamilton Rating Scale for Depression (HAM-D17) [31], Major Depression Inventory (MDI) [31], World Health Organization-Five Well-Being Index (WHO-5) [31], Geriatric Depression Scale-15 (GDS-15) [32], and the Edinburgh Postnatal Depression Scale (EPDS) [34]. In spite of the fact that the majority of included studies (five studies) confirmed the positive effect of supplementation of vitamin D for the psychological measure of depression [29,32,33,34,35], for three studies, the positive influence was not supported [30,31,36]. Moreover, it should be emphasized that the positive influence of vitamin D was confirmed for the studies with a shorter study duration—those conducted for 8 weeks [29,32,33,34] and one conducted for 12 weeks [35]—while it was not confirmed for the other studies conducted for 12 weeks [30,31] and for one conducted for 6 months [36]. Last but not least, it should be mentioned that in the studies for which vitamin D deficiency was indicated within the inclusion criteria [30,36], and in those for which it was indicated within the exclusion criteria [31], the positive influence of applied vitamin D doses was not confirmed, but such confirmation was obtained only for the mixed populations.

The risk of bias assessment for studies included to a systematic review, conducted using the revised Cochrane risk-of-bias tool for randomized trials, is presented in Table 5. For the majority of included studies, a medium risk of bias was indicated [29,30,31,32,33,34], while for only two studies, a high risk of bias was defined [35,36] due to deviations from the intended interventions and in measurement of the outcome [35,36], as well as for one study, also arising from the randomization process and due to missing outcome data [35]. This corresponds the fact that in the indicated studies, the control group was not treated with a placebo, but was just without vitamin D treatment [35,36]. For the studies with a medium risk of bias, this resulted mainly from the randomization process and in the selection of the reported result [29,30,31,32,33,34], but for two studies was also due to deviations from the intended interventions [30,31]. The studies for which the high risk of bias was indicated were within those supporting the positive influence of the supplementation of vitamin D for the psychological measure of depression [35], and those not supporting [36]. However, based on the conducted assessment, it should be emphasized that there are only four studies supporting the positive influence of vitamin D supplementation for the psychological measure of depression of medium risk of bias [29,32,33,34], while two studies with a medium risk of bias did not support it [30,31].

## 4. Discussion

The conducted systematic review did not allow to formulate unambiguous conclusions about influence of the supplementation of vitamin D for the psychological measure of depression, as the various observations were formulated within studies, which was not associated with the studied group, sample size, or studied effect. Despite that, for two studies of major depressive disorders, the positive effect of vitamin D was indicated [29,35]; one of them was defined as associated with the high risk of bias [35], so it does not allow us to extrapolate. It may indicate that there was only one study of postpartum depression defined as associated with a medium risk of bias that confirmed the positive influence of vitamin D, which may be promising [34].

Taking into account the observations from the included studies, the potential mechanism of influence of vitamin D on depression must be considered. However, the current knowledge about the effect of vitamin D on neuronal brain functioning and behaviors is based on animal studies, as little is known based on human studies so far [37]. The association between vitamin D and depression is probably associated with the fact that vitamin D receptors are not only present, but even widespread, in the hippocampus and other brain areas implicated in depression [38]. At the same time, vitamin D metabolites are able to cross the blood–brain barrier, which allows them to act there [39], as well as in vitro and in vivo studies in animal models revealed that vitamin D deficiency may influence the shape or function of the hippocampal development [40]. Similarly, the human studies indicated that vitamin D deficiency is associated with decreased brain tissue and hippocampal volume, detected during brain magnetic resonance imaging (MRI) [41].

In general, vitamin D is a neuroactive steroid which contributes to the expression of neurotransmitters with its regulation and neuroimmunomodulation, antioxidant production, and various neurotrophic factors [40]. It may also upregulate genes involved in the synthesis of tyrosine hydroxylase, being an enzyme involved in the synthesis of catecholamines [42] that is potentially involved in depression development and treatment [43]. All indicated actions of vitamin D may participate in the mechanism of depression treatment and prevention, but the question arises as to whether they are significant enough to participate in treatment, or only in prevention. The role of vitamin D is generally indicated as protective [37] by potential reducing of the negative effects of dopaminergic toxins, possibly by increasing glial cell-line-derived neurotrophic factor and affecting serotonin transmission in the brain, linking dopaminergic and serotonergic systems [44,45]. Similarly, the preventive role of vitamin D is confirmed by the recent meta-analysis of RCTs by Xie et al. [46], which indicated its beneficial impact on the incidence of depression.

At the same time, for the treatment, the results are not unambiguous as various studies provide diverse observations and conclusions. While more human studies are needed to make a conclusion, the quality of the necessary studies should be emphasized, as not only RCTs should be conducted, but they should be rigorously planned and executed.

Moreover, it should be emphasized that there is also a need to assess the influence of vitamin D on depression in children and adolescents, which should be the aim of the further studies. So far, it may be indicated that—similarly as for adults—the results are not unambiguous here. In the RCT by Libuda et al. [47] for adolescents with vitamin D deficiency and at least mild depression, the study failed to prove a vitamin D supplementation effect on self-rated depression, but parents of the treated adolescents reported fewer depressive symptoms in their progeny. 

As revealed within the presented systematic review, the quality of included studies is the major problem. An important problem is also the need for placebo-based studies (instead of studies with control group without placebo), which are especially important for such a condition as depression, as the effectiveness of the placebo is proven to increase the effect of cognitive–behavioral therapy in depressive patients [48]. Taking this into account, not receiving any treatment within the study may contribute to seeking other therapeutic options, or to reduced effectiveness of the standard therapy as a result of the feeling of deprivation [49]. The other limitation of the conducted study results from a heterogeneity of included studies, using the various psychological measures of depression and studying various populations. As the conducted systematic review presents only the limited number of studies published so far, while included studies presented various studied groups, outcomes, and psychological measures, more studies are necessary to deepen the observations. Moreover, since the studies assessed a wide range of possible psychological measures, meta-analysis was impossible [50], so only the systematic review was conducted.

## 5. Conclusions

Based on the conducted assessment, there are only four studies supporting the positive influence of vitamin D supplementation for the psychological measure of depression of medium risk of bias, while two studies with a medium risk of bias did not support it. Taking this into account, the conducted systematic review is not a strong confirmation of the effectiveness of vitamin D supplementation in the treatment of depression.

## Figures and Tables

**Table 1:** The patient, intervention/exposure, comparator, outcome, and study design (PICOS) criteria.

PICOS Criterion	Inclusion	Exclusion
Population	Adult patients with depression diagnosed	Pregnant women, patients with any concurrent physical disease or disability, patients with concurrent eating disorders, patients with concurrent intellectual disabilities
Intervention/exposure	Vitamin D oral supplementation of known dose	Multiple nutrients supplementation
Comparison	Compared with control group	No comparison with control group without vitamin D supplementation
Outcome	Depression monitored	No valid mental health outcome measure applied
Study design	Randomized Controlled Trials (RCTs) published as articles in peer-reviewed journals	Studies not published in English, animal model studies

**Table 2:** The general descriptions of the studies and studied populations within studies included to a systematic review.

Ref.	Authors, Year	Country/Detailed Location	Studied Population	Period of the Study
[29]	Khoraminya et al., 2013	Iran/Tehran	Patients with major depressive disorder from the Roozbeh Psychiatry Hospital, Tehran University of Medical Sciences, Tehran	From November 2010 to December 2011
[30]	Marsh et al., 2017	United States of America (USA)/Massachusetts	Patients with bipolar depression and vitamin D deficiency from central Massachusetts, USA	From June 2013 to April 2015
[31]	Hansen et al., 2019	Denmark/Esbjerg, Odense and Svendborg	Patients with depressive episode from the mood disorder clinic in the Region of Southern Denmark	From November 2010 to June 2014
[32]	Alavi et al., 2019	Iran	Older patients with moderate to severe depression from three psychiatric clinics	From March 2016 to February 2017
[33]	Kaviani et al., 2020	Iran/Tehran	Patients with mild to moderate depression referred to the outpatient clinics of Baharloo Hospital	From May 2018 to June 2019
[34]	Amini te al., 2020	Iran/Ahvaz	Female patients with postpartum depression from the outpatient clinic of Ahvaz Jundishapur University of Medical Sciences	From June to November 2017
[35]	Alghamdi et al., 2020	Saudi Arabia/Jeddah	Patients with major depressive disorder from the psychiatry clinic at the King Abdulaziz University Hospital	Not specified (3 months)
[36]	Zhu et al., 2020	China/Anhui	Patients with depression, anxiety and low 25(OH)D levels recruited through advertisements from Anhui Mental Health Center	From November 2015 to September 2019

**Table 3:** The descriptions of the studied populations within studies included to a systematic review.

Ref.	Number of Participants (Female)	Age (Mean with SD/Range)	Inclusion Criteria	Exclusion Criteria
[29]	40 (34)	38.1 ± 10.1 years (vitamin D group) 39.6 ± 8.3 years (control group)	Inclusion: 18–65 years; diagnosis of major depressive disorder without psychotic features based on DSM-IV criteria; score of ≥15 on the 17-item HDRS; not taking any antidepressant or dietary supplements during the previous 2 months; being free from other psychiatric diagnoses, significant medical illnesses, or suicidal thoughts	Exclusion: substance abuse; pregnancy; lactation; occurrence of important adverse effects from medications
[30]	33 (16) for baseline	45.2 ± 13.3 years (vitamin D group) 43.3 ± 12.9 years (control group)	Inclusion: 18–70 years; bipolar disorder spectrum diagnosis (bipolar I, II, not otherwise specified); experiencing depressive symptoms rating ≥7 on the MADRS; having psychiatric care provider; if on psychotropic medication—a stable dose for the previous 2 weeks and remained on a current medication regime; serum 25-hydroxyvitamin D levels ≤ 30 ng/mL or insufficient	Exclusion: insulin dependent diabetes mellitus; liver and kidney diseases; parathyroid disorder; disorders of vitamin D metabolism; abnormal parathyroid hormone, calcium, or phosphorous level; taking Vitamin D replacement therapy; fat digestion disorder; gastrointestinal surgery; active suicidality; acute psychosis; active substance use <3 months
[31]	62 (47) for baseline	39.6 ± 13.5 years (vitamin D group) 38.7 ± 11.4 years (control group)	Inclusion: 18–65 years; depressive episode according to the ICD-10—mild to severe depression	Exclusion: bipolar affective disorder; any form of schizophrenia; tuberculosis; sarcoidosis; pregnancy; intake of more than 400 IU vitamin D daily; known allergy/intolerance to the content of the capsules; women in potential of childbearing if they did not utilize effective contraception; serum 25(OH)D < 10 nmol/L or > 100 nmol/L; serum calcium (ionized) > 1.40 mmol/L; estimated glomerular filtration rate (eGFR) < 60 mL/min/1.73 m^2^; serum phosphate < 1.50 mmol/L (females) or < 1.60 mmol/L (males aged 18–49 years) or < 1.35 mmol/L (males > 49 years), or serum PTH > 9.2 pmol/L
[32]	78 (39)	68.7 ± 7.0 years (vitamin D group) 67.0 ± 6.3 years (control group)	Inclusion: >60 years; GDS score > 5—moderate to severe depression; treatment for depression; Iranian citizenship; ability to speak Farsi and answer the questions	Exclusion: history of mental illness other than depression; history of physical disability; uncooperative; severe stress such as hospitalization or death of relatives
[33]	56 (50)	43.1 ± 9.2 years (vitamin D group) 42.8 ± 8.0 years (control group)	Inclusion: 18–60 years; BDI-II score of 13–29—mild to moderate depression	Exclusion: other psychiatric disease according to the psychiatrist’s assessments; history of heart infarction, angina pectoris, stroke, kidney stones, high blood pressure, liver disease, hyperparathyroidism; pregnancy and/or lactation; women <50 years not receiving adequate contraception; consuming nutritional supplements containing vitamin D from two months prior to the intervention
[34]	76 (76)	26.9 ± 1.0 years (vitamin D and calcium group) 29.2 ± 1.4 years (vitamin D group) 28.9 ± 1.6 years (control group)	Inclusion: women; 18–45 years; EPDS score >12—postpartum depression score; postpartum period from 1 to 6 months; BMI < 35 kg/m^2^	Exclusion: serum vitamin D value >75 nmol/L; birth abnormalities; taking contraceptive agents; endocrine disorders; history of severe depression and/or other mental disorders; using antidepressants; serum calcium concentration >2.65 mmol/L; intake of vitamin D and calcium supplements during previous 6 months; history of diabetes, renal failure, kidney stones, gastrointestinal diseases
[35]	62 (missing data)	41.5 ± 1.8 years	Inclusion: 18–65 years; diagnosis of major depressive disorder based on DSM-V criteria	Exclusion: abnormal PTH level; renal or hepatic impairment
[36]	106 (78)	46.3 ± 9.7 years (vitamin D group) 43.3 ± 13.7 years (control group)	Inclusion: 18–60 years; diagnosis of major depressive disorders according to DSM-V; Han Chinese ethnicity; serum 25(OH) D ≤ 75 nmol/L	Exclusion: other concurrent psychiatric disorders defined in DSM-V; substance use disorders; current severe physical conditions; pregnancy; lactation

BDI-II—Beck Depression Inventory-II; BMI—body mass index; DSM-IV—Diagnostic and Statistical Manual of Mental Disorders; DSM-V—Diagnostic and Statistical Manual of Mental Disorders; EPDS—Edinburgh Postnatal Depression Scale; GDS—Geriatric Depression Scale; HDRS—Hamilton Depression Rating Scale; ICD-10—the International Classification of Diseases; MADRS—Montgomery-Åsberg Depression Rating Scale; PTH—parathyroid hormone.

**Table 4:** The descriptions of the vitamin D supplementation and the assessment of depression status accompanied by the summary of conclusions formulated within studies included to a systematic review.

Ref.	Vitamin D Supplementation Dose Regimen	Vitamin D Supplementation Duration	Psychological Measure of Depression	Summary of Conclusions *
[29]	1500 IU of vitamin D3 daily	8 weeks	24-item Hamilton Depression Rating Scale (HDRS) 21-item Beck Depression Inventory (BDI)	Confirming
[30]	1500 IU of vitamin D3 daily	12 weeks	Montgomery-Åsberg Depression Rating Scale (MADRS)	Not confirming
[31]	2800 IU of vitamin D3 daily	12 weeks	Hamilton Depression Rating Scale-17 (HAMD-17) Major Depression Inventory (MDI) World Health Organization-Five Well-Being Index (WHO-5)	Not confirming
[32]	50,000 IU of vitamin D3 weekly	8 weeks	Geriatric Depression Scale-15 (GDS-15)	Confirming
[33]	50,000 IU of vitamin D3 biweekly	8 weeks	Beck Depression Inventory-II (BDI-II)	Confirming
[34]	50,000 IU of vitamin D3 biweekly	8 weeks	Iranian Edinburgh Postnatal Depression Scale (EPDS)	Confirming
[35]	50,000 IU of vitamin D3 weekly	12 weeks	Beck Depression Inventory (BDI)	Confirming
[36]	1600 IU of vitamin D3 daily	6 months	Hamilton Depression Rating Scale-17 (HAMD-17)	Not confirming

* The summary of conclusions defined as confirming (if confirmed positive influence of vitamin D supplementation for the psychological measure of depression) or not confirming (if not confirmed positive influence of vitamin D supplementation for the psychological measure of depression).

**Table 5:** The risk of bias assessment for studies included to a systematic review, conducted using the revised Cochrane risk-of-bias tool for randomized trials.

Ref.	Domain 1	Domain 2	Domain 3	Domain 4	Domain 5	Overall Bias
[29]	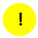	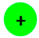	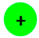	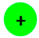	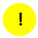	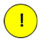
[30]	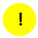	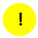	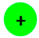	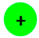	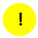	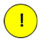
[31]	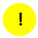	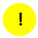	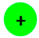	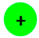	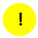	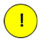
[32]	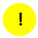	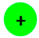	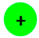	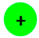	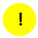	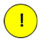
[33]	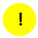	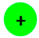	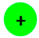	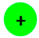	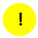	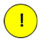
[34]	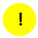	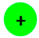	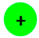	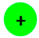	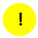	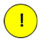
[35]	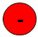	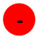	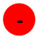	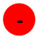	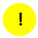	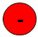
[36]	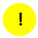	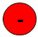	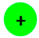	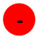	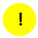	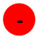

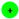
—Low risk of bias; 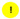
—Some concerns associated with risk of bias 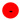
—High risk of bias; Domains: 1—arising from the randomization process; 2—deviations from the intended interventions; 3—missing outcome data; 4—measurement of the outcome; 5—selection of the reported result.

## Supplementary Materials

The following are available online at https://www.mdpi.com/article/10.3390/nu15040951/s1, Table S1: The detailed electronic search strategy applied for the systematic review within PubMed or Web of Science databases. Table S2: The observations and conclusions formulated within studies included to a systematic review.
