# Supplementary material for: Effect of Vitamin D Supplementation on Depression in Adults: A Systematic Review of Randomized Controlled Trials (RCTs)
Source: Nutrients. 2023 Feb 14;15(4):951. doi: 10.3390/nu15040951 (PMC9963956; doi:10.3390/nu15040951)
Supplement: Supplementary file 1 [file nutrients-15-00951-s001.zip › nutrients-2167018-supplementary.pdf]

nutrients

MDPI

Systematic Review

Effect of Vitamin D Supplementation on Depression in Adults: A Systematic Review of Randomized Controlled Trials (RCTs)

Dominika Guzek [1], Aleksandra Kołota [2], Katarzyna Lachowicz [2], Dominika Skolmowska [2], Małgorzata Stachoń [2] and Dominika Głąbska [2,*]

Supplementary Table S1. The detailed electronic search strategy applied for the systematic review within PubMed or Web of Science databases.

Database	The applied full electronic search strategy
PubMed	(((mental health[T/A] OR mental disorders[T/A] OR mental disorder[T/A] OR psychological distress[T/A] OR mood disorders[T/A] OR depression[T/A] OR suicidal[T/A] OR suicide[T/A] OR anxiety[T/A] OR well-being[T/A] OR wellbeing[T/A] OR quality of life[T/A] OR self esteem[T/A] OR self-esteem[T/A] OR self efficacy[T/A] OR self-efficacy[T/A] OR resilience[T/A] OR empowerment[T/A] OR social participation[T/A] OR mental capital[T/A] OR life skills[T/A] OR emotional[T/A] OR psychology[T/A] OR psychosocial[T/A] OR psychiatry[T/A])) AND (vitamin D[T/A] OR vitamin D2[T/A] OR vitaminD3[T/A] OR D2[T/A] OR D3[T/A] OR ergocalciferol[T/A] OR cholecalciferol[T/A] OR 25-hydroxyvitamin D[T/A] OR 3-epi-25hydroxyvitaminD[T/A] OR calcitriol[T/A] OR dihydroxycholecalciferol[T/A])) NOT (animal NOT (animal AND human)[MeSH Terms])
Web of Science	(TS=("vitamin D" OR "vitamin D2" OR "vitamin D3" OR "D2" OR "D3" OR "ergocalciferol" OR "cholecalciferol" OR "25-hydroxyvitamin D" OR "3-epi-25 hydroxyvitamin D" OR "calcitriol" OR "dihydroxycholecalciferol") AND TS=("mental health" OR "mental disorders" OR "mental disorder" OR "psychological distress" OR "mood disorder" OR "depression" OR "suicidal" OR "suicide" OR "anxiety" OR "well-being" OR "wellbeing" OR "quality of life" OR "self esteem" OR "self-esteem" OR "self efficacy" OR "self-efficacy" OR "resilience" OR "empowerment" OR "social participation" OR "mental capital" OR "life skills" OR "emotional" OR "psychology" OR "psychosocial" OR "psychiatry") NOT TS=("animal" NOT ("animal" AND "human")))

T/A – Title/Abstract

Supplementary Table S2. The observations and **conclusions** formulated within studies included to a systematic review.

Ref.	Observations	Conclusions
[29]	The vitamin D + fluoxetine combination was significantly better than fluoxetine alone from the fourth week of treatment.	The vitamin D + fluoxetine combination was superior to fluoxetine alone in controlling depressive symptoms.
[30]	MADRS score decreased significantly in both placebo (mean = 6.42 (95% CI [2.28 to 10.56]) and vitamin D groups (mean = 9.54 (95% CI[3.51 to 15.56]) (p = 0.031), but there were no differences between treatment groups (time by treatment interaction estimate: 0.29, t(23) = 0.14, p = 0.89).	There was no significant difference reduction in depressive symptoms. Vitamin D3 supplementation vs placebo did not improve reduction in mood elevation or anxiety symptoms.
[31]	No significant reduction in depression was seen after vitamin D supplementation compared to placebo at Hamilton (18.4–18.0; p = 0.73 at 12 weeks).	Vitamin D supplementation did not provide a reduction in symptom score among patients with depression.
[32]	The depression score decreased from 9.25 to 7.48 in vitamin D group (p = 0.0001), while there was a non-significant increase in depression score in placebo group. The multiple regression analysis	Vitamin D supplementation can improve the depression score in persons aged 60 and over.

	showed that the vitamin D group and the score of depression before study were the variables that could explain 81.8% of depression score after intervention.	
[33]	Following intervention, significant changes were observed in the intervention group compared to the controls: BDI scores decreased (-11.75±6.40 vs. -3.61±10.40, $p = 0.003$).	Eight-week supplementation with 50,000 IU/2wks vitamin D significantly improved depression severity.
[34]	The postpartum depression score had more reduction in the vitamin D + calcium and vitamin D groups than that of the placebo group (−1.7 ± 3.44, −4.16 ± 5.90 and 0.25 ± 2.81, respectively; $p = 0.008$). The effect of vitamin D on the postpartum depression score was larger when vitamin D was given alone than given together with calcium ($p = 0.042$ and $p = 0.004$, respectively).	Vitamin D may be effective in improving the clinical symptoms of postpartum depression.
[35]	The results showed significant gender differences; female patients showed the most improvement in their depressive symptoms after 3-month vitamin D supplementation. Females with moderate, severe, and extreme depression had significantly lower BDI scores after vitamin D treatment ($p < 0.05$). Among males, only those diagnosed with severe depression showed significant improvement in their BDI scores ($p < 0.05$).	Vitamin D supplementation may ameliorate symptoms of MDD, particularly in females.
[36]	In this clinical population, no significant difference in depression symptoms was detected between vitamin D group and control group at both baseline and at the endpoint of our study. The HAMD-17 scores did not change significantly between vitamin D and control groups from baseline to endpoint ($p > 0.05$).	Vitamin D supplementation could improve the anxiety symptoms but not depressive symptoms in depressive patients with low vitamin D level after the 6-month intervention.

MADRS – Montgomery-Åsberg Depression Rating Scale; BDI – Beck Depression Inventory; MDD – Major Depressive Disorder; HAMD-17 – Hamilton Depression Rating Scale-17.
